# Supplementary material for: Network pharmacology and integrative bioinformatics analyses identify PDE1A as a key target of pirfenidone in idiopathic pulmonary fibrosis
Source: PLoS One. 2026 Apr 10;21(4):e0342991. doi: 10.1371/journal.pone.0342991 (PMC13068261; doi:10.1371/journal.pone.0342991)
Supplement: Attachment — Submitted filename: Authors Response Letter.docx [file pone.0342991.s002.docx]

Dear Editor,

We are extremely grateful to the editors and reviewers for the opportunity they have given us to revise the manuscript. We believe that through these supplements and revisions, our research can better meet the publication standards of PLOS ONE. We look forward to your further feedback and hope that the revised manuscript can be accepted for publication.

Response to Reviewer’s Comment:

Reviewer #1：Changes made according to reviewer 1 are highlighted in blue in the article.

1. The In-silico analysis presented in this manuscript falls short of established standards. While computational approaches are valuable, they require proper validation and detailed methodology to support conclusions.

Reply: Thank you for your valuable comments on our manuscript. We attach great importance to the issue you pointed out regarding the need for more rigorous validation and detailed methodological support for In silico analysis. We are fully aware of the deficiencies in the methodological description and result verification of the computer simulation part in the original manuscript. For this reason, in the revised draft:

(1) Clarify the technical details of molecular docking, including the acquisition of protein structures, determination of binding sites, and other technical details.

(2) Add experimental verification (MST). To enhance the reliability of the calculation results, we have newly added the micro thermal Migration (MST) experiment to verify the binding relationship between PDE1A and candidate compounds. The experimental results are consistent with the computational predictions, supporting the conclusion of molecular docking at the experimental level.

(3) In the molecular docking results, the analysis of the interaction between proteins and compounds has been added and the interaction has been visualized.

These results suggest that Pirfenidone may exert its biological effects by binding specifically to PDE1A. We have described in detail in the text the parameter Settings of molecular docking, the scoring system and the analysis of binding sites to ensure the transparency and repeatability of this part of the research. The modifications in the article are as follows: **Interaction analysis of Pirfenidone and PDE1A: MD and MST**

The binding patterns of PDE1A (Fig. 5A) and pirfenidone (Fig. 3B) were predicted through molecular docking (Fig. 5C). The docking results indicated that the binding energy between PDE1A and pirfenidone was -6.4 kcal/mol (Table 1), and they formed extensive interactions. Specifically, the side chains of amino acids H219 and H263 of PDE1A formed hydrogen bonds with pirfenidone. In addition, ligands also have π-π stacking and π- cation interactions with H219 residues. These bonding and non-bonding interactions work in synergy, enhancing the binding stability of the complex (Fig. 5D). In this study, we conducted a detailed analysis of the interaction between Pirfenidone and PDE1A through Microscale Thermophoresis (MST) technology. The experimental conditions are as follows: Ligand Concentration: 1000 µM to 0.0305 µM; Excitation Power: 100%; MST Power: 40%; Temperature: 25.0°C; Kd: 1.5097E-05; Kd Confidence: ± 2.5858E-06; Signal to Noise: 19.171163. The MST results show that a signal-to-noise ratio greater than 5 indicates a cutoff, while a ratio greater than 12 indicates high-quality data. Moreover, the protein mobility slows down with increasing analyte concentration. The fitting curve is S-shaped, exhibiting clear upper and lower plateau phases and strong concentration dependence; this indicates specific binding (Fig. 5E-F). The dissociation constant (Kd) is 15.1 μM, indicating relatively strong binding affinity in the in vitro molecular interaction between the protein and small molecules.

**Tanble 1 Molecular docking scoring item**

| **Combination mode** | **Affinity (kcal/mo1)** | **RMSD(1.b.)** | **RMSD(u.b.)** |
| --- | --- | --- | --- |
| **1** | -6.4 | 0 | 0 |
| 2 | -6.3 | 3.025 | 3.737 |
| **3** | -6.3 | 2.388 | 5.651 |
| **4** | -6.3 | 2.444 | 5.564 |
| **5** | -6.3 | 2.818 | 4.441 |
| **6** | -6.3 | 3.439 | 4.844 |
| **7** | -6.2 | 2.063 | 5.665 |
| **8** | -6.1 | 1.781 | 2.105 |
| **9** | -6.0 | 2.100 | 3.173 |

**Fig. 5 The experimental results of MD and MST**. A: Structural diagram of protein PDE1A; B: Pirfenidone structure diagram; C: The docking structure diagram of Pirfenidone and PDE1A; D: 3D diagram of the interaction between Pirfenidone and PDE1A (affinity -6.4 kcal/mol), The green rod-like structure is a small molecule; The brown rod-like structure is the residue of the protein structure, Yellow dotted line: Hydrogen bond Pink dotted line: π-πinteraction; Orange dotted line: π-cation; E: Each curve in the figure is a real-time recording curve of fluorescence intensity in a capillary tube, which records the variation of fluorescence intensity over time in the temperature gradient. Purple represents the initial average signal F0, red represents the average signal F1 of the time period selected for the fitting graph, and the homogenized fluorescence signal Fnorm is the thousandth ratio of F1/F0. As the heating begins, the fluorescently labeled protein surges towards the surrounding low-temperature area. The density of the fluorescent protein per unit space decreases, so the Fnorm value will decrease. After the heating ends, the temperature recovery gradient disappears and the Fnorm value rebounds; F: Fnorm fitting graph.

The methods in the article have been re-supplemented:

**Molecular Docking (MD)**

Macromolecular docking is one of the important methods of molecular simulation. It is a type of computer simulation. Its essence is the recognition process between two or more molecules. This process involves shape and spatial complementarity as well as energy compatibility between molecules. In the field of molecular modeling, if two molecules can form a stable complex, molecular docking can predict their binding mode. Furthermore, the binding strength can be evaluated using a scoring function based on this mode.

The software adopted this time is AutoDock Vina. The algorithm of AutoDock Vina is the Monte Carlo search algorithm based on local perturbation. The method of ligand docking with PDE1A (phosphodiesterase 1A, an enzyme involved in cellular signaling) is semi-flexible docking, that is, the ligand is flexible (the rotatable bond angle can rotate freely), while the PDE1A protein is rigid (remains unchanged). The 3D structure file of the receptor protein was downloaded from the PDB, and the protein structure was completed and optimized. If there is no crystal structure of the experimental data, AlphaFold3 is used to predict the 3D structure of the receptor protein (PDE1A: UniProt ID P5475). Download the 3D structure file of the small molecule compound from the PubChem database and perform structure optimization on the compound (Pirfenidone: PubChem ID 40632). If the structure file is unavailable, the StoneMIND Collector is used to draw the compound, generate the 3D structure, and then perform energy minimization. The active pockets of proteins are determined by integrating information provided by users, annotations from the UniProt database, and AI predictions. This project uses KVFinder[21] to predict the active pockets of proteins, determines the active sites of the proteins, and performs molecular docking using AutoDock Vina. After processing and optimizing the docking data, the results were analyzed using Discovery Studio Visualizer.

**MicroScale Thermophoresis (MST)**

MicroScale Thermophoresis (MST) is a powerful technique for quantifying interactions between biomolecules. MST combines precise fluorescence detection with sensitive and flexible thermophoresis to provide a powerful and rapid method for measuring intermolecular interactions. Specifically, during the MST experiment, the sample is heated by an infrared laser to generate a microscopic temperature gradient. Then, the directional movement of molecules is monitored and quantified through covalently bound fluorescent dyes or the autofluorescence of tryptophan. The application scope includes interactions involving small molecules, proteins, and protein complexes.

**(1) Protein labeling (NTA dye)**

Take a new clean 1.5 mL centrifuge tube, add 2 µL of the newly prepared RED-tris-NTA second-generation dye and 98 µL of PBS-T, and gently pipette and mix evenly with a pipette to obtain a dye solution with a final concentration of 100 nM. Then add 100 µL of 200nM protein sample. After wrapping the centrifuge tubes with tin foil, incubate them at room temperature in the dark for 30 minutes in the dark.

After the protein labeling step is completed, centrifuge at 4 ° C and 15,000 g for 10 minutes, and take the supernatant into a new centrifuge tube.

**(2) Protein labeling fluorescence intensity detection (Pretest)**

After fluorescence labeling, 200 μL of protein was obtained. 10μL was taken out and diluted 3 times with Assay Buffer. Open the sample chamber, take out the tray, draw the diluted protein sample with a capillary tube and place it in the corresponding slot of tray 1.2. Input protein sample information and Assay Buffer information; Select the capillary type; Excitation Power: Select Auto; MST-Power selects Medium.

**(3) Binding Affinity determination**

Dilute the analyte to twice the maximum final loading concentration of 100μL using the Assay Buffer. Take two eight-row samples, make labels 1-16, and add 10μL of Assay Buffer to tubes 2-16 respectively; Add 20μL of the prepared analyte to tube 1, and use a pipette to draw 10 μL. Start the 2-fold gradient dilution successively in tubes 2 to 16. Then, starting with a pipette, add 10μL of protein to each tube and pipette to mix well. After the mixed sample is incubated at room temperature for 15 minutes, it is aspirated with a capillary tube and loaded onto the sample tray. Then, click "Start" on the software to begin the experiment.

Data analysis was conducted after repeating the steps of dilution - protein mixing - sample loading for a total of three times.

2. The authors state they downloaded structures of seven differentially expressed genes from the RCSB Protein Data Bank but fail to provide the specific PDB IDs. This omission prevents proper assessment and reproducibility of the work.

Reply: Thank you very much for your attention and valuable suggestions to our manuscript. When we were writing the method section, the information was incorrect due to a typo. This is entirely due to our negligence. Thank you for pointing out this problem. We have made modifications in the method section and supplemented the specific PDB IDs. The following is the revised description: The software adopted this time is AutoDock Vina. The algorithm of AutoDock Vina is the Monte Carlo search algorithm based on local perturbation. The method of ligand docking with PDE1A (phosphodiesterase 1A, an enzyme involved in cellular signaling) is semi-flexible docking, that is, the ligand is flexible (the rotatable bond angle can rotate freely), while the PDE1A protein is rigid (remains unchanged). The 3D structure file of the receptor protein was downloaded from the PDB, and the protein structure was completed and optimized. If there is no crystal structure of the experimental data, AlphaFold3 is used to predict the 3D structure of the receptor protein (PDE1A: UniProt ID P5475). Download the 3D structure file of the small molecule compound from the PubChem database and perform structure optimization on the compound (Pirfenidone: PubChem ID 40632).

3. The manuscript claims that a docking binding energy of less than −5 kcal/mol indicates strong binding affinity. This threshold is an oversimplification of molecular docking. Proper docking studies typically include comprehensive analysis of binding poses and conformations. The assertion that -5 kcal/mol represents strong binding is arbitrary without contextual validation.

Reply: Thank you very much for your attention and valuable suggestions to our manuscript. We have deleted the expression "A binding energy less than -5 kcal/mol is considered strong binding" in the original text and changed it to a more cautious description, such as: "Generally, a binding energy less than -5 kcal/mol can be regarded as having certain binding potential, but the specific binding ability still needs to be verified in combination with other experiments." We supplemented the multi-angle analysis of the docking results, including specific interactions such as hydrogen bonds, hydrophobic interactions, and π-π packing, and combined with visual images to present the binding mode between ligands and targets. The modifications in the article are as follows:

**Interaction analysis of Pirfenidone and PDE1A: MD and MST**

The binding patterns of PDE1A (Fig. 5A) and pirfenidone (Fig. 3B) were predicted through molecular docking (Fig. 5C). The docking results indicated that the binding energy between PDE1A and pirfenidone was -6.4 kcal/mol (Table 1), and they formed extensive interactions. Specifically, the side chains of amino acids H219 and H263 of PDE1A formed hydrogen bonds with pirfenidone. In addition, ligands also have π-π stacking and π- cation interactions with H219 residues. These bonding and non-bonding interactions work in synergy, enhancing the binding stability of the complex (Fig. 5D). In this study, we conducted a detailed analysis of the interaction between Pirfenidone and PDE1A through Microscale Thermophoresis (MST) technology. The experimental conditions are as follows: Ligand Concentration: 1000 µM to 0.0305 µM; Excitation Power: 100%; MST Power: 40%; Temperature: 25.0°C; Kd: 1.5097E-05; Kd Confidence: ± 2.5858E-06; Signal to Noise: 19.171163. The MST results show that a signal-to-noise ratio greater than 5 indicates a cutoff, while a ratio greater than 12 indicates high-quality data. Moreover, the protein mobility slows down with increasing analyte concentration. The fitting curve is S-shaped, exhibiting clear upper and lower plateau phases and strong concentration dependence; this indicates specific binding (Fig. 5E-F). The dissociation constant (Kd) is 15.1 μM, indicating relatively strong binding affinity in the in vitro molecular interaction between the protein and small molecules.

4. No positive control was included in the docking experiments. For reliable docking protocols, re-docking of co-crystal ligands (whenever possible) with RMSD calculations below 2Å is typically used to validate methodology.

Reply: Thank you very much for your attention and valuable comments on our manuscript. However, due to the lack of co-crystalline data of PDE1A protein, re-docking cannot be carried out. In order to improve the accuracy of the molecular docking method, we have made the following adjustments.

At present, there is no relevant co-crystalline structure data for PDE1A (UniProt number: P54750), nor is there experimental crystal structure information of the protein. Meanwhile, there is no recognized positive control drug yet. Therefore, re-docking verification based on the binding sites of positive compounds cannot be carried out.

2. In this study, AlphaFold3 was used to predict the three-dimensional structure of PDE1A (with a pTM score of 0.75), and KVFinder was utilized to predict potential compound binding sites. Based on this binding site, molecular docking was carried out using AutoDock Vina. Subsequently, the interaction between the protein and the ligand was analyzed in detail with the help of Discovery Studio Client, and the visualization display of the binding mode was achieved by using PyMOL.

5. The manuscript lacks critical details regarding amino acid residues involved in the interaction with the ligand and the forms of interactions. A thorough docking analysis should include both bonding and non-bonding interactions and an examination of key contact points between the ligand and receptor.

Reply: Thank you to the reviewers for your valuable comments. We supplemented the detailed analysis of the molecular docking results and further clarified the key interactions between ligands and receptor proteins. Calculated by AutoDock Vina, the binding free energy of PDE1A to the compound was -6.4 kcal/mol, showing a good binding potential. The specific binding mode analysis indicates that the ligand has formed stable hydrogen bond interactions with the key amino acid residues H219 and H263 of PDE1A. In addition, ligands also have π-π stacking and π- cation interactions with H219 residues. These bonding and non-bonding interactions work in synergy, enhancing the binding stability of the complex. Detailed visual analysis of the relevant binding sites has been supplemented in the revised draft to enable readers to understand the interaction mechanism more intuitively.

6. The authors should perform in vitro validation of the binding or conduct molecular dynamics simulations of at least 200ns to assess the stability of the protein-ligand complex over time.

Reply: Thanks for the reviewer's comments! To verify the interaction between PDE1A and Prifenidone, the MST technique was newly added for in vitro interaction tests. The Kd value of the interaction between PDE1A and Prifenidone was obtained as 15.1 μM, which proved the interaction between PDE1A and Prifenidone and was consistent with the result of molecular docking. Given that we have conducted reliable experimental verification, long-term molecular dynamics simulations have not been carried out in this study for the time being. However, we fully recognize the value of MD simulation in verifying the stability of the combination. In future work, we will further explore the application of MD simulation in this system.

7. Discuss the limitations of computational approaches. The study relies on limited computational evidence without sufficient validation or detailed analysis.

Reply: Thank you to the reviewers for your valuable comments. We are fully aware that the computational methods in this study have certain limitations. Molecular docking, in particular, is mainly based on a rigid protein model and empirical scoring function. Therefore, it is difficult to comprehensively reflect the dynamic process and thermodynamic details of protein-ligand interactions. To address the insufficiency of computational evidence, we conducted the microscale thermophoresis (MST) experiment in the revised draft, measured the binding affinity between PDE1A and the ligand, and provided important experimental verification for the computational results. Furthermore, we have added a detailed explanation of the limitations of the calculation method in the discussion section, clearly stating that the results need to be comprehensively interpreted in combination with the experimental data. The article discussion is supplemented as follows:

Nevertheless, this study efficiently uncovers potential binding sites and key amino acid residues. However, we acknowledge that this computational approach has certain limitations. First, AutoDock treats proteins primarily as rigid bodies during molecular docking, allowing only the flexible rotational degrees of freedom of ligands. This limitation can make it difficult to accurately capture true binding conformations, especially for protein targets with obvious conformation-inducing effects. Furthermore, the AutoDock scoring function is primarily based on empirical energy terms, including simplified energy calculation models such as van der Waals forces, electrostatic forces, hydrogen bonding, and hydrophobic interactions. While these energy terms are valuable for rapidly screening large numbers of ligands, the scoring function cannot comprehensively account for complex factors such as the effect of solvent on proteins and ligands in aqueous environments, conformational entropy changes, and adjustments in protein flexibility. Therefore, the predicted binding free energy often contains significant errors and does not accurately reflect the actual binding affinity. Using molecular docking technology, we computationally identified potential interactions between PDE1A and pirfenidone. We subsequently performed an MST experiment and determined a K_D value of 15.1 μM for the interaction between PDE1A and pirfenidone. This value was in agreement with the molecular docking results and confirmed the interaction. Further studies, including mutagenesis, structural biology, and in vivo validation, are required to confirm the therapeutic relevance of PDE1A inhibition by pirfenidone.

Reviewer #2：Changes made according to reviewer 2 are highlighted in blue in the article.

1. structures of seven differentially expressed genes were obtained from the RCSB Protein Data Bank. Please mention the PDB ids) (PDB, https://www.rcsb.org/) and99

saved in PDB format.

Reply: Thank you very much for your attention and valuable suggestions to our manuscript. When we were writing the method section, the information was incorrect due to a typo. This is entirely due to our negligence. Thank you for pointing out this problem. We have made modifications in the method section and supplemented the specific PDB IDs. The following is the revised description: The software adopted this time is AutoDock Vina. The algorithm of AutoDock Vina is the Monte Carlo search algorithm based on local perturbation. The method of ligand docking with PDE1A (phosphodiesterase 1A, an enzyme involved in cellular signaling) is semi-flexible docking, that is, the ligand is flexible (the rotatable bond angle can rotate freely), while the PDE1A protein is rigid (remains unchanged). The 3D structure file of the receptor protein was downloaded from the PDB, and the protein structure was completed and optimized. If there is no crystal structure of the experimental data, AlphaFold3 is used to predict the 3D structure of the receptor protein (PDE1A: UniProt ID P5475). Download the 3D structure file of the small molecule compound from the PubChem database and perform structure optimization on the compound (Pirfenidone: PubChem ID 40632).

2. CB-Dock2 conducts blind docking. Why not active site-specific docking?

Reply: Thank you very much for your attention and valuable suggestions on our manuscript. We re-conducted the molecular docking analysis. Since there is currently no co-crystal structure of the PDE1A protein and ligand available, we first used the KVFinder tool to predict its possible binding pockets. Based on these predictions, binding pockets with larger volume and higher druggability were selected as candidate binding regions. Targeted molecular docking analysis was then performed using AutoDock Vina to obtain more reasonable binding patterns and interaction information.

3. Before docking the co-crystal ligand should be redocked and rmsd with diagram should be mentioned in the supplementary material.

Reply: We fully understand and agree with the importance of Re-docking and RMSD analysis of molecular docking tags. However, at present, there are no high-resolution co-crystalline structure data of PDE1A and small molecule ligands, so it is impossible to conduct standard re-docking verification of the system studied in this study. To enhance the reliability of the docking results, we have taken the following measures:

1. Use AlphaFold3 to predict the protein structure of PDE1A (pTM=0.75).

2. Use KVFinder to predict possible binding pockets and avoid non-specific docking

3. Use AutoDock Vina for targeted docking with target restrictions and conduct structural analysis of the binding mode

4. In the experimental section, the MST experiment was supplemented, which verified the actual binding ability between the protein and the ligand from an experimental perspective, further supporting the credibility of the docking results.

The modifications in the article are as follows: **Interaction analysis of Pirfenidone and PDE1A: MD and MST**

The binding patterns of PDE1A (Fig. 5A) and pirfenidone (Fig. 3B) were predicted through molecular docking (Fig. 5C). The docking results indicated that the binding energy between PDE1A and pirfenidone was -6.4 kcal/mol (Table 1), and they formed extensive interactions. Specifically, the side chains of amino acids H219 and H263 of PDE1A formed hydrogen bonds with pirfenidone. In addition, ligands also have π-π stacking and π- cation interactions with H219 residues. These bonding and non-bonding interactions work in synergy, enhancing the binding stability of the complex (Fig. 5D). In this study, we conducted a detailed analysis of the interaction between Pirfenidone and PDE1A through Microscale Thermophoresis (MST) technology. The experimental conditions are as follows: Ligand Concentration: 1000 µM to 0.0305 µM; Excitation Power: 100%; MST Power: 40%; Temperature: 25.0°C; Kd: 1.5097E-05; Kd Confidence: ± 2.5858E-06; Signal to Noise: 19.171163. The MST results show that a signal-to-noise ratio greater than 5 indicates a cutoff, while a ratio greater than 12 indicates high-quality data. Moreover, the protein mobility slows down with increasing analyte concentration. The fitting curve is S-shaped, exhibiting clear upper and lower plateau phases and strong concentration dependence; this indicates specific binding (Fig. 5E-F). The dissociation constant (Kd) is 15.1 μM, indicating relatively strong binding affinity in the in vitro molecular interaction between the protein and small molecules.

**Tanble 1 Molecular docking scoring item**

| **Combination mode** | **Affinity (kcal/mo1)** | **RMSD(1.b.)** | **RMSD(u.b.)** |
| --- | --- | --- | --- |
| **1** | -6.4 | 0 | 0 |
| 2 | -6.3 | 3.025 | 3.737 |
| **3** | -6.3 | 2.388 | 5.651 |
| **4** | -6.3 | 2.444 | 5.564 |
| **5** | -6.3 | 2.818 | 4.441 |
| **6** | -6.3 | 3.439 | 4.844 |
| **7** | -6.2 | 2.063 | 5.665 |
| **8** | -6.1 | 1.781 | 2.105 |
| **9** | -6.0 | 2.100 | 3.173 |

**Fig. 5 The experimental results of MD and MST**. A: Structural diagram of protein PDE1A; B: Pirfenidone structure diagram; C: The docking structure diagram of Pirfenidone and PDE1A; D: 3D diagram of the interaction between Pirfenidone and PDE1A (affinity -6.4 kcal/mol), The green rod-like structure is a small molecule; The brown rod-like structure is the residue of the protein structure, Yellow dotted line: Hydrogen bond Pink dotted line: π-πinteraction; Orange dotted line: π-cation; E: Each curve in the figure is a real-time recording curve of fluorescence intensity in a capillary tube, which records the variation of fluorescence intensity over time in the temperature gradient. Purple represents the initial average signal F0, red represents the average signal F1 of the time period selected for the fitting graph, and the homogenized fluorescence signal Fnorm is the thousandth ratio of F1/F0. As the heating begins, the fluorescently labeled protein surges towards the surrounding low-temperature area. The density of the fluorescent protein per unit space decreases, so the Fnorm value will decrease. After the heating ends, the temperature recovery gradient disappears and the Fnorm value rebounds; F: Fnorm fitting graph.

4. There are some differences between freeing binding energy and binding affinity. Please correct as because at the beginning "Molecular Docking" binding affinity was mentioned!

Reply: Thank you, reviewers, for your meticulous corrections. We have carefully examined the expressions regarding "binding energy" and "binding affinity" throughout the text, especially the description at the beginning of the "molecular docking" section. We found that some word choices were inaccurate. "Binding free energy" is a calculated estimate reflecting the thermodynamic stability of intermolecular interactions, while "binding affinity" usually refers to the experimentally measured molecular binding strength, such as the dissociation constant (Kd). Although the two are related, they are not completely equivalent. We have clarified these distinctions in the revised draft, avoiding directly equating the calculated binding free energy with binding affinity. Additionally, experimental data, including affinity measurements such as Kd determined by MST, were used to supplement the verification of the calculation results, thereby supporting our conclusions more comprehensively.

5. How docking binding energy of less than −5 kcal/mol was considered indicative of strong binding affinity?? is there any control drug used or how this threshold was decided>

Reply: Thank you for the valuable comments of the reviewers. Thank you for the valuable suggestions put forward by the reviewers. We recognize the advantages of the MM-PBSA method in the calculation of binding free energy, especially that it can evaluate the thermodynamic stability of protein-ligand complexes more accurately based on molecular dynamics. However, this study mainly conducts molecular docking based on AutoDock Vina. The binding conformations generated by AutoDock Vina have not undergone molecular dynamics simulation. Therefore, the MM-PBSA method cannot be directly applied to calculate the binding free energy at present. For this reason, in the revised draft, we have added a detailed analysis of the molecular docking binding mode and verified the binding affinity of the protein to the ligand in combination with the data from the micro thermal migration (MST) experiment.

6. I suggest to conduct mm-PBSA as because docking-based mm-GBSA is not accurate to calculate free binding energy!

Reply: Thank you for the valuable suggestions put forward by the reviewers. We recognize the advantages of the MM-PBSA method in the calculation of binding free energy, particularly its ability to evaluate the thermodynamic stability of protein-ligand complexes more accurately based on molecular dynamics. However, this study mainly performs molecular docking using AutoDock Vina. The binding conformations generated by AutoDock Vina have not undergone molecular dynamics simulation. Therefore, at present, the MM-PBSA method cannot be directly applied to calculate the binding free energy. For this reason, in the revised draft, we have added a detailed analysis of the molecular docking binding mode. We also verified the binding affinity of the protein to the ligand based on data from the MST experiment.

Thank you again for your support and understanding.

Best wishes!

Yanyan Ke
