# Supplementary material for: Network pharmacology and integrative bioinformatics analyses identify PDE1A as a key target of pirfenidone in idiopathic pulmonary fibrosis
Source: PLoS One. 2026 Apr 10;21(4):e0342991. doi: 10.1371/journal.pone.0342991 (PMC13068261; doi:10.1371/journal.pone.0342991)
Supplement: Attachment — Submitted filename: Authors Response Letter- PONE-D-24-60633R2.docx [file pone.0342991.s003.docx]

03058490558

Reviewer #1：Changes made according to reviewer 1 are highlighted in blue in the article.

1. Regarding binding energy reviewer 1 also highlighted the same. I suggest that the authors may take 3 months time use good software such as Autodock Vina, SwissDock or any other molecular docking software and perform the docking correctly.

Reply: Thank you for your valuable comments on our manuscript.

We used software such as Discovery studio2019, Schrodinger2021, and Swiss-dock to re-perform molecular docking. The connection result is as follows:

| Software | Binding energy |
| --- | --- |
| Autodock vina | -6.4 kcal/mol |
| Discovery studio2019 | -8.124 kcal/mol |
| Schrodinger2021 | -4.41349 kcal/mol |
| Swiss-dock | -6.2662 kcal/mol |

This is the best conformation given by different software:

2. As mm-gbsa did not provided good results, question is arising about the authenticity of the data. I suggest to conduct "Consensus molecular docking" using multiple docking software and identify the best pose.

Reply: Thank you very much for your attention and valuable suggestions to our manuscript. We have answered this question in Question 1

3. Take an extension of 3-4 months and try to conduct 200 to 500 ns Molecular dynamic simulation to understand the Protein-Ligand interaction dynamics.

Reply: Thank you very much for your attention and valuable suggestions to our manuscript. We conducted a new molecular dynamics simulation. The simulation video is available on PDE1A.mp4 and has been modified as follows in the article:

We conducted the analysis using Discovery Studio Visualizer: Through the analysis of the docking results, hydrogen bond interactions were formed between his219 and HIS263 of the PDE1A protein and the small molecule, while HIS219 and the small molecule formed Pi-Pi and Pi-cation interactions. The van der Waals forces provided by the amino acids around the small molecule jointly stabilized the small molecule in the binding pocket (Figure 6A). The first frame of the dynamic simulation trajectory was selected as a reference to calculate the root mean square deviation (RMSD) of Cα atoms. In the initial stage (0-20 ns), the RMSD of both rapidly increased, indicating that the system had adjusted from the initial conformation to the equilibrium state. Subsequently, the RMSD fluctuation range of the complex was mainly around 0.6-0.8 nm, showing a relatively stable trend, although there were still small fluctuations. The RMSD fluctuation range of the protein monomer is slightly wider, approximately 0.6-1.0 nm, and it shows an upward trend in the later stage of the simulation (160-200 ns). Overall, the RMSD of the complex seems to stabilize earlier than that of the protein monomers, and the fluctuation range is also smaller. The overall fluctuations of the protein monomers and complexes were within the acceptable range, indicating stable binding (Figure 6B). According to the RMSF comparison between wt and complex (amino acids 5A away from the ligand molecule), among the amino acids at positions 260-269, 330-333, 366-367, and 384-388 that constitute the ligand-binding pocket, the loop region composed of 260-269 and 330-333 fluctuates. In the protein-ligand complex, the ligand remained stable near the binding pocket throughout the 200ns kinetic simulation, indicating that the ligand formed a stable complex structure with the protein under these simulation conditions (Figure 6C). In this study, the Rg of the protein-ligand complex was lower than that of the protein monomer, reflecting that the persistent presence of the ligand in the binding pocket enhanced the compactness of the protein through local structural optimization and non-polar interactions (Figure 6D). In this study, the reduction of the complex SASA indicates that ligand binding buried part of the protein surface, reducing solvent accessibility. The stability of the pockets further confirmed that this change was caused by the ligand occupying the exposed area, highlighting the crucial role of hydrophobic interactions in maintaining the stability of the complex (Figure 6E). In this study, the charts show that the initial binding may rely on hydrogen bonds, but the number of hydrogen bonds decreased significantly after 100 ns. The disappearance of hydrogen bonds may be related to the dissociation of ligands at binding sites, conformational changes, or the rearrangement of hydrogen bond donors/acceptors. The reduction of Pairs within 0.35 nm further supports the reduction of close contact between ligands and proteins. The bifurcation of the red line (H-bond) and the blue line (Pairs within 0.35 nm) in the figure indicates that some pairs may still exist but no longer meet the geometric requirements of hydrogen bonds (Figure 6F). As shown in the figure, the free energy morphology diagram was drawn with the RMSD and Rg trajectories at the last 10ns of the 200ns simulation. In the 2D diagram, the blue area indicates the lowest free energy point of protein-ligand binding, meaning that the protein-ligand complex at this site is the most stable conformation with the lowest free energy point throughout the simulation process (Figure 6G-I).

**Fig. 6 The experimental results of MD and MST**. A: Proteins dock with small molecules; B: RMSD result graph; C: RMSF Fluctuation analysis; D: Circumferential radius result; E: Solvent accessible surface area; F: Number of hydrogen bonds

; G-I: Free energy topography.

In this study, gmx_MMPBSA was used to calculate the free energy with Gromacs trajectories and topological files. The total binding energy of each complex was contributed by different components, including VDWAALS, EEL, EPB, ENPOLAR, GGAS and GSOLV. According to the results of RMSD, The trajectory of the last 10ns period after ligand stabilization was selected for MM/PBSA analysis. From the calculation results, it can be known that the average binding free energy of the two is -14.67 kcal/mol (Table 2).

**Table 2 Protein binding free energy with small molecules (MM/PBSA analysis)**

| **Energy Component** | **Average** |
| --- | --- |
| **ΔVDWAALS** | **-25.62** |
| **ΔEEL** | **-13.92** |
| **ΔEPB** | **27.71** |
| **ΔENPOLAR** | **-2.84** |
| **ΔGGAS** | **-39.54** |
| **ΔGSOLV** | **24.87** |
| **ΔTOTAL** | **-14.67** |

Corrections are added in page14, line 252.

4. I have a doubt whether the author chosen the right protein target or not! Authors should also think about it and if found true then change it accordingly. With current results it is difficult to agree with the conclusion.

Reply: Thank you very much for your attention and valuable comments on our manuscript. We have re-conducted the molecular dynamics experiment, and the results have been replied in question 3.

5. Reply to point 5 and 6 is same, whereas the context of the points were different!

Reply: Thank you to the reviewers for your valuable comments. We conducted molecular dynamics simulations of at least 200 nanoseconds to evaluate the temporal stability of protein-ligand complexes. The simulation results can be seen in the video file PDE1A.mp4.
