# Supplementary material for: Network pharmacology and integrative bioinformatics analyses identify PDE1A as a key target of pirfenidone in idiopathic pulmonary fibrosis
Source: PLoS One. 2026 Apr 10;21(4):e0342991. doi: 10.1371/journal.pone.0342991 (PMC13068261; doi:10.1371/journal.pone.0342991)
Supplement: Attachment — Submitted filename: Reviewer Response Letter- R4- 16-01-2026.docx [file pone.0342991.s005.docx]

**Response to Reviewer Comments**

We sincerely thank the reviewer for the constructive and detailed comments. All observations have significantly improved the quality, clarity, and reproducibility of our manuscript. We have carefully revised the manuscript as per suggestion and tried our best to incorporate all recommended changes.

**Reviewer Comment:**

The study is relevant but lacks reproducibility and methodological rigor. Additional methodological detail, MD validation, improved references, identifiers, and data/code availability are required.

**Response:** We have now substantially expanded the methodological descriptions, added detailed molecular dynamics (MD) analyses, corrected references, and included explicit dataset identifiers. All the generated data is available in the public repositories and the accession numbers are mentioned in the manuscript. These modifications have been fully incorporated into the revised manuscript.

**Reviewer Comment:** Provide step-by-step methodological detail.

**Response:** A comprehensive workflow description has been added, covering gene identification, target prediction, enrichment analysis, docking procedures, and validation steps. This has been incorporated throughout the Methods section.

**DEG criteria**

We have now specified thresholds, adjusted p-values, and the Benjamini–Hochberg method for multiple testing. This information is added.

**GEO processing**

Platform IDs and normalization approach covariates have been described in detail. Implemented in Methods.

**Tools/versions**
Exact software, versions, and packages (where applicable) used for analysis and data processing have been added to ensure reproducibility. Incorporated in Methods.

**Justification for PDE1A selection**

We expanded the explanation for prioritizing PDE1A among the seven DEGs based on functional importance, enrichment relevance, and reported links with fibrosis. Added where applicable.

**Pose validation**

Pose clustering method, key residues, and selection criteria have been added. Incorporated at appropriate position in the manuscript.

**Functional Enrichment**

We now specify ontology categories (GO BP/CC/MF), KEGG and Reactome usage, significance cutoffs, background gene set, and multiple-testing corrections. Included in manuscript.

**Statistics**

Sample sizes, test types, normality checks, and two-sided assumptions are now clearly stated. Incorporated in manuscript.

**Reviewer Comment:** Some references were not aligned and formatting needed correction.

**Response:** All references have been rechecked and reformatted.

**Reviewer Comment:** Docking alone is insufficient; MD simulations must be performed and reported.

**Response:** We fully agree. We have now performed molecular dynamics simulations using GROMACS and Ligand RMSD, Protein RMSF, Hydrogen-bond and hydrophobic interaction occupancies, Water-bridge analysis, MM/PBSA binding energies (mean ± SD) and other necessary analyses have been performed. All MD methodology and results have been integrated into the manuscript, including new figures and tables.

**Regulatory approval**

We have now specified the agency and year of pirfenidone approval (FDA 2014; EMA 2011) with primary citations.

**MD integration**

The MD findings are now integrated at appropriate positions in the manuscript.

We have added PDB IDs and UniProt IDs for PDE1A, GEO dataset IDs with GSM groupings, platform IDs, and Links of the utilized public repositories.

All identifiers are now clearly listed in the manuscript.

**Response:** In compliance with the reviewer’s request, we have explained the GEO preprocessing docking protocols with tool names, parameters and versions; analyze MD trajectories and mentioned the results in the manuscript at appropriate positions, topologies, and analysis code. DOI included in manuscript where needed. This ensures full transparency and reproducibility.

All requested analyses have been performed and added, including RMSD, RMSF, MM/PBSA binding energies, and other necessary analyses.

These results are presented in the revised Results.

Reviewer #3 Comment:

The RMSF analysis could be strengthened by a residue-specific interpretation and quantitative comparison between apo and ligand-bound systems.

**Response:** The RMSF analysis has been revised to include residue-specific interpretation of regions 260–269, 330–333, 366–367, and 384–388, with explicit discussion of their secondary structure context and proximity to the binding pocket. Additionally, a comparison of RMSF values between apo and pirfenidone-bound systems performed and reduced fluctuation was observed in pirfenidone-bound systems. These revisions clarify the ligand-induced stabilization of PDE1A dynamics without requiring additional simulations, as suggested. The results have been mentioned in the manuscript at appropriate positions.

We are grateful for the reviewer’s detailed and rigorous comments. All recommended revisions have been thoroughly implemented, and the manuscript has been significantly strengthened in terms of methodology, reproducibility, scientific depth, and clarity. We have incorporated each modification into the revised manuscript as requested.

Thank you for your consideration.
