# Supplementary material for: Network pharmacology and integrative bioinformatics analyses identify PDE1A as a key target of pirfenidone in idiopathic pulmonary fibrosis
Source: PLoS One. 2026 Apr 10;21(4):e0342991. doi: 10.1371/journal.pone.0342991 (PMC13068261; doi:10.1371/journal.pone.0342991)
Supplement: Attachment — Submitted filename: Reviewer Response Letter- R5- 28-01-2026.docx [file pone.0342991.s006.docx]

**Response to Reviewer Comments- R5**

We are very grateful for the valuable time and constructive comments provided by both the editor and the reviewers. All observations have significantly improved the quality, clarity, and reproducibility of our manuscript. We have carefully addressed all reviewers’ comments and made every effort to incorporate all recommended changes.

**Reviewer Comment:** The manuscript can be accepted once all trajectory data and the code used to generate the related figures are made publicly available. For example, the complete trajectory dataset may be uploaded to Zenodo or a similar repository, with the corresponding access link included in the Data Availability section.

**Response:** We thank the reviewer for this valuable suggestion. In response, all trajectory data and the complete code used to generate the related figures have now been made publicly available. The full dataset has been deposited in the Zenodo repository and can be accessed via the following DOI: <https://doi.org/10.5281/zenodo.18387883>. The corresponding access link has also been included in the Data Availability section of the online portal as well as revised manuscript.
